# Airway Symptoms and Biological Markers in Nasal Lavage Fluid in Subjects Exposed to Metalworking Fluids

**DOI:** 10.1371/journal.pone.0083089

**Published:** 2013-12-31

**Authors:** Louise Fornander, Pål Graff, Karin Wåhlén, Kjell Ydreborg, Ulf Flodin, Per Leanderson, Mats Lindahl, Bijar Ghafouri

**Affiliations:** 1 Occupational and Environmental Medicine, Department of Clinical and Experimental Medicine, Faculty of Health Sciences, Linköping University, Linköping, Sweden; 2 Centre of Occupational and Environmental Medicine, County Council of Östergötland, Linköping, Sweden; 3 Clinic of Otorhinolaryngology, County Hospital Ryhov, Jönköping, Sweden; 4 Rehabilitation Medicine, Department of Medical and Health Sciences, Faculty of Health Sciences, Linköping University, Pain and Rehabilitation Centre, County Council of Östergötland, Linköping, Sweden; University of Liverpool, United Kingdom

## Abstract

**Backgrounds:**

Occurrence of airway irritation among industrial metal workers was investigated. The aims were to study the association between exposures from water-based metal working fluids (MWF) and the health outcome among the personnel, to assess potential effects on the proteome in nasal mucous membranes, and evaluate preventive actions.

**Methods:**

The prevalence of airway symptoms related to work were examined among 271 metalworkers exposed to MWF and 24 metal workers not exposed to MWF at the same factory. At the same time, air levels of potentially harmful substances (oil mist, morpholine, monoethanolamine, formaldehyde) generated from MWF was measured. Nasal lavage fluid was collected from 13 workers and 15 controls and protein profiles were determined by a proteomic approach.

**Results:**

Airway symptoms were reported in 39% of the workers exposed to MWF although the measured levels of MWF substances in the work place air were low. Highest prevalence was found among workers handling the MWF machines but also those working in the same hall were affected. Improvement of the ventilation to reduce MWF exposure lowered the prevalence of airway problems. Protein profiling showed significantly higher levels of S100-A9 and lower levels of SPLUNC1, cystatin SN, Ig J and β2-microglobulin among workers with airway symptoms.

**Conclusions:**

This study confirms that upper airway symptoms among metal workers are a common problem and despite low levels of MWF-generated substances, effects on airway immune proteins are found. Further studies to clarify the role of specific MWF components in connection to airway inflammation and the identified biological markers are warranted.

## Introduction

Workers in the metal industry are exposed to a wide range of substances that can affect their health. One common type of exposure comes from metal working fluids (MWFs), which are used in the metal processing to cool and lubricate, as well as preventing corrosion and removing generated metal chips and swarf from the machine site. MWFs are divided into four classes (straight, soluble, semi-synthetic and synthetic) depending on the amount of oil they contain. Except for oil and water, the MWF usually contains a range of additives, such as biocides, surfactants, anti-oxidants and corrosion inhibitors. Each additive on its own may negatively affects the workers' health [1]. During metal processing, workers are exposed to aerosols that may generate airway symptoms such as coughing, rhinitis and wheezing. Furthermore, asthma, hypersensitivity pneumonitis and chronic bronchitis have been described in MWF exposed metal workers [2]–[4]. Beside airway symptoms, skin problems are not unusual and MWFs have been shown to cause occupational allergic contact dermatitis [5]. Over time there has been a shift from oil-based MWFs to water-based MWFs and thereby the health problems connected to oil-based MWFs has decreased.

Different factors contribute to the work environment generated by MWF aerosols. Even though many irritative substances generated from MWFs are known, the direct cause for the health problems in the factories are often unclear. Studies have shown that the aerosol may consist of particles in respirable size fractions, and depending on the composition contain different chemical compounds such as formaldehyde, alkanolamines, triazoles and volatile organic compounds [6]–[10]. Although exposure through inhalation is a major route, skin uptake may also be substantial, as shown for ethanolamines [11]. Along with increased use of water-based MWFs more attention has been given to the importance of microbes and microbial pro-inflammatory components, such as endotoxin [12]. Over time the MWFs are likely to be contaminated with microbes, even though biocides are used [13]–[14]. For example, Pseudomonas rods and different types of mycobacteria have been identified and linked to the occurrence of hypersensitive pneumonitis [14]–[15].

Prior to the performed study, several metal factories were visited in the southeast region of Sweden to assess the use of MWFs and to estimate the occurrence of health problems by interviews and a preliminary questionnaire. In total, 29 factories with over 1500 employees were visited. This pre-survey showed that 70% of the factories had personnel with airway and skin problems suspected to be related to both oil-based and water-based MWFs. One large factory had, according to the occupational health care records, a history of skin problems due to oil-based MWF. After introducing oil mist separators in the machineries and shifting to water-based MWF, an increased number of complaints from the personnel about airway irritation were reported, including those not working at machines containing MWFs. Simultaneously, the complaints regarding skin problems declined. This factory was chosen for a more detailed study with the aims to investigate the association between exposures from water-based MWFs and the health outcome among the personnel, to study changes in the nasal mucous membranes due to the work environment by biomonitoring the protein profile, and suggest and evaluate preventive actions.

## Subjects and Methods

### Chemicals

Sodium dodecyl sulfate (SDS), iodoacetamide, DTT, CHAPS, 2,5-dihydroxybenzoic acid, acetonitrile, trifluoroacetic acid, Phorbol 12-myristate 13-acetate (PMA, no. P8139), lipopolysaccharide (LPS, from Salmonella enterica, serotype minnesota) and bovine serum albumin (BSA) were all purchased from Sigma-Aldrich (St. Louis, MI, USA). 40% acrylamide solution, 2% bis-acrylamide solution, TEMED, Tween 20 and ammonium persulfate were obtained from Bio-Rad Laboratories (Hercules, CA, USA). Culture media RPMI 1640, foetal bovine serum (FBS) and penicillin/streptomycin were purchased from Gibco. Antibodies and standards for the TNF-α and IL-1β ELISA were from Diaclone (Human TNF-α Eli-pair, Diaclone, Besancon, France). The ECL+ detection kit (Western Blotting Detection System) came from GE Healthcare, (Little Chalfont, UK). IPG buffer pH 3–10 NL, IPGs NL 3–10, and dry strip cover fluid were acquired from GE Healthcare and porcine trypsin was from Promega (Madison, WI, USA). The calibration mixture for peptide mass fingerprinting; des-Arg1-bradykinin, angiotensin I, Glu1-fibrinopeptide B, neurotensin, adrenocorticotropic hormone (ACTH, clip 1–17), ACTH (clip 18–39), ACTH (clip7–38) with masses: 904.4681, 1296.6853, 1570.6774, 1672.9175, 2093.0867, 2465.1989, 3657.9294, respectively, were purchased from Applied Biosystems (Foster City, CA, USA).

### Study design

A cross-sectional descriptive study of a metal factory using MWF in the southeast region of Sweden was performed. As start, a main questionnaire (MM 040 NA) regarding experienced health status, work environment and indoor climate was sent out to all workers [16]. 295 subjects answered the questionnaire with an answering frequency of 78%. The subjects were categorized in two groups depending on MWF exposure. One group (n = 271) working in a large hall (about 20000 m^2^) used for metal processing and containing about 30 machines with MWF, and a second group (n = 24) working in a separate hall, of approximately the same size as the processing hall, used for assembly and without exposure to MWF. The group working in the processing hall was further divided into those who were directly exposed to MWF (operated machines that used MWFs, n = 102) and those who were indirectly exposed to MWF (not operated machines that used MWFs, n = 169). A reference group consisting of school and office personnel (n = 4780) was used as controls [16]. At the same time as the questionnaire, several environmental measurements were made in the factory hall to study health problems in relation to exposure.

From the answers, 15 subjects with (n = 9) or without (n = 6) airway symptoms related to work and MWF exposure were recruited by the occupational health care for more detailed analysis. To exclude allergies as cause for experienced airway symptoms, or skewness between groups, ImmunoCAP® Rapid Asthma/Rhinitis Adult (Thermo Fisher Scientific, Uppsala, Sweden), a blood test for Ig E profile, was used. Only a few subjects were found to be Ig E-positive, mostly for grass pollen, and there was no difference in Ig E-profiles between the groups. The 15 exposed subjects were all men, non-smokers with a mean age of 50±10 years. At the same time, 15 controls were recruited outside of the industrial environment, with no known health problems, to be investigated in similar manner. They were all men, non-smokers and with a mean age of 46±13 years. All subjects were subjected to nasal lavage fluid (NLF) sampling for protein profiling, assessment of nitric oxide (NO) from exhaled breath and a questionnaire to monitor health status at sampling time. Written consent was obtained from all participants. The study was approved by the local ethical board at Linköping University (Dnr: M39-09).

Preventive measures were suggested by an occupational hygienist. These include transferring polluted air from the machines out of the processing hall and not recycled, improvements of the ventilation in the processing hall, not to open the machines before the oil mist had been ventilated out of the machines and reduce the use of compressed air to clean the products in the processing hall as this resulted in a spread of MWF mist. To monitor the effect of preventive actions at the factory after the first main questionnaire, a follow-up questionnaire regarding airway symptoms was used to analyze the health status among the personnel at two time points. The first follow-up questionnaire was sent out 12 months after the main questionnaire and just prior to rebuilding the ventilation of the machines. After this work, the processing air, instead of being recycled back to the main hall, was rerouted out of the building. The second follow-up questionnaire was sent out 6 months after the rebuilding was completed.

### Exposure measurements

All measurements at the metal factory were performed in the processing hall where MWFs were used, with the exception of formaldehyde that was measured in the separate mounting hall where no MWF were used. Morpholine and monoethanolamine were measured using silica tubes coupled to AirCheck 2000 air sampling pump (SKC Inc., Eighty Four, PA, USA) with a flow rate of 0.5 l/min for 25 min. The samples were analyzed by liquid chromatography with a mass selective detector by the Finnish Institute of Occupational Health (Turku, Finland) according to Henriks-Eckerman [11]. Dust and oil mist were collected using glass fiber filters coupled to AirCheck 2000 air sampling pump with a flow rate of 2 l/min for 6 h for dust and 5 h for oil mist. Gravimetric analysis was done at Occupational and Environmental Medicine (Linköping, Sweden) for dust and Occupational and Environmental Clinic (Örebro, Sweden) for oil mist. Endotoxin was collected using polycarbonate filters with a pore size of 0.45 µm coupled to AirCheck 2000 air sampling pumps with a flow rate of 1.5 l/min for 3 h and analyzed by Eurofins (Pegasus Lab., Uppsala, Sweden) with a limulus amebocyte lysate test. The flow rate of the pumps was, before use, calibrated with BIOS DryCal DC-Lite (Mesa Labs., Lakewood, CO, USA). Formaldehyde was sampled both stationary and person bound with UME^X^ 100 Passive Samplers for formaldehyde from SKC for 6 h. The samples were analyzed with liquid chromatography after desorption with acetonitrile at Occupational and Environmental Medicine (Linköping, Sweden). At time for follow-up questionnaires, formaldehyde was sampled stationary again.

### NO measurement

Exhaled NO was measured with NIOX Mino (Aerocrine, Solna, Sweden) prior to nasal lavage fluid sampling. A flow rate of 50 ml/s was used according to the manufacturer's instruction at the time of measurement. All subjects exhaled against a positive counter pressure of 1.013 hPa to prevent contamination of the sample with nasal air. A filter was used to avoid mucous to enter the NIOX Mino and a reference value between 5 to 25 ppb was considered normal [17].

### Nasal lavage fluid sampling

Nasal lavage fluid was retrieved by introducing 15 ml of 0.9% saline solution to the nasal cavity by a catheter and maintained there for 5 minutes before recovered. The nasal lavage fluid was filtered through a mesh and mucous and cells were immediately removed by centrifugation (700 rcf). Samples were kept on ice during transport to the lab (3 h) where they were divided in aliquots and stored at −70°C until use. Protein concentration was determined according to Bradford [18]. Two samples, one from a subject with airway symptoms and one from a subject without airway symptoms, contained very low protein concentrations and were not used for protein profile analysis. The nasal lavage sampling was made outside of pollen season.

### Nasal lavage fluid analysis

#### Two-dimensional gel electrophoresis and image analysis

The 2-DE analysis was performed with a horizontal 2-DE setup (IPGphor and Multiphor, GE Healthcare) as described previously [19]. Briefly, in the first dimension, 50 µg proteins were applied by in-gel rehydration for 12 h using low voltage (30 V) on pH 3–10 non-linear IPG's. Focusing of the proteins were run for 38 000 Vh at a maximum voltage of 8000 V. The second dimension (SDS-PAGE) was made by transferring the proteins to gradient home cast gels on GelBond PAG film (0.5*180*245 mm, T: 11–18%, C: 1.5%, 33–0% glycerol) running at 30 mA for about 5 h. The gels were silver stained and analyzed using a 3.2 megapixel CCD (Charged-Coupled Device) camera digitizing at 53 µm resolution (VersaDoc 4000 MP) in combination with analysis software PDQuest Advanced 8.0.1 (both from Bio-Rad Laboratories, CA, USA). The different images were evaluated using an approach previously described [20]. Briefly, a match set was created and the gel with most detected spots was used as the master gel and the other images were then matched to the master gel. The amount of protein in a spot was expressed as background corrected optical density (OD). In order to correct for differences in total silver stain intensity between different 2-DE images, the amounts of the individual protein spots were normalized to the total protein intensity on the gels. Thereby %OD for all protein spots was generated and was evaluated for differences between the groups. Although the protein expressions vary between individuals the variation in the quantitative determinations of a protein spot is <10% with this approach [20]. After statistical analysis, protein spots which expressed significant differences between groups were further analyzed and identified by mass spectrometry.

#### Identification of proteins with mass spectrometry

Protein spots were excised from the gel and destained with 30 mM potassium ferricyanide/50 mM sodium thiosulfate, followed first by incubation with 200 mM ammonium bicarbonate and then with 100% acetonitrile to dehydrate the gel piece. Trypsin, 10 mg/ml, was added to the samples, incubated overnight in 37°C and then dried in a speed-vac. The peptides were reconstituted in 0.1% trifluoroacetic acid and mixed 1∶1 with matrix (2,5-dihydroxybenzoic acid, 0.02 mg/ml) as described by Ghafouri [19]. The samples were applied onto the target plate (V700666 REV.C, Applied Biosystems) followed by peptide acquisition with a MALDI-TOF MS (Voyager-DE PRO, Applied Biosystems, Foster City, CA, USA) using a 337 nm N_2_ laser, positive mode, reflector mode, delayed extraction and instruments settings a defined earlier [19]. Spectra were obtained in mass range 700–3600 m/z with close external mass calibration using a standard peptide mixture and internal calibration using known trypsin autolysis peaks (m/z 842.5100, 2211.1046). Data Explorer™ version 4.0 was used for processing spectra and Protein Prospector v 5.10.11 and MS Fit to analyze searches in Swiss-Prot database. During Swiss-Prot searches restrictions was set on species (human), mass tolerance (±50 ppm), cystein modification by carbamidomethylation and maximum missed cleavage by trypsin to one. The 50–100 most abundant peaks were used for database searches and identification of proteins with peptide mass fingerprinting.

#### Western blot analysis of SPLUNC1 and S100-A9 levels

NLF samples containing 15 µg total proteins from each subject were separated by SDS-PAGE gel electrophoresis (5–20%) on Mini-protean II electrophoresis cell (Bio-Rad). The proteins were then transferred to a PVDF membrane, blocked and incubated with primary antibodies against SPLUNC1 (goat polyclonal, R&D Systems, MN, USA) and S100-A9 (mouse monoclonal, ab24111, Abcam, Cambridge, UK) in TTBS with 2% non-fat dried milk overnight. Membranes were washed with TTBS followed by incubation with HRP-conjugated secondary antibody against SPLUNC1 (sheep anti-goat IgG, SIGMA, MI, USA) and S100-A9 (goat-anti mouse IgG, Bio-Rad) for 1 h. The antigen/antibody conjugate was visualized using chemiluminescence ECL solution (GE healthcare) and CCD camera (Bio-Rad). The blots were further quantified in gray-scale image by Image Lab Software (Bio-Rad). Optical density (OD) was used to quantify expressed proteins and the results are presented as OD×10^3^.

#### Analysis of MPO and IL-1β in nasal lavage fluid

Determination of MPO was made with sandwich ELISA, similar to a method described by Chang *et al* [21]. Rabbit anti-MPO (Meridian Life Science, Saco, ME, USA) was used as coating antibody, and mouse anti-human MPO-HRP from Abcam (Cambridge, UK) was used as detection antibody. The measurement of MPO was made after 15 minutes incubation with ECL Plus on a chemiluminescence plate reader (Lumistar, BMG Labtechnologies, Offenburg, Germany). The within and between day coefficient of variation was 4.8% and 7.9% respectively. IL-1β was analysed with ELISA using matched capture and detection antibodies (IL-1β ELI-Pair Set, #851.610.010) as described previously [22]. Measurements of chemiluminescence were performed as described above.

#### MWF-mediated formation of tumor necrosis factor-alpha by cells in vitro

Blood from healthy volunteers were collected in heparinized tubes (BD Vacutainer, Plymouth, UK) and used within two hours. Samples of new and used MWF (nine parts of water and one part of mineral oil containing sodium sulfonate, ethanolamine and methylene-bis-oxazine) taken from the metal industry were diluted in 0.9% NaCl to 0.001, 0.01, 0.1 and 1%. In addition, lipopolysaccharide (1 and 0.1 ng/ml) was used as a positive control. Each sample (200 µl) was mixed with 50 µl of whole blood and incubated for 4 h in 37°C and 5% CO_2_ before centrifugation and analysis of TNF-α in the supernatants.

THP-1 cells (a human monocyte cell line) were maintained in RPMI 1640 supplemented with 5% FBS and 100 U/ml penicillin/streptomycin, in a humid atmosphere containing 5% carbon dioxide, at 37°C. Before experiment, cells were allowed to differentiate into a macrophage-like phenotype. This was done by seeding 5×10^4^ cells in 200 µl culture medium with 50 nM PMA into wells in a 96-well plate (Costar 3610, Corning incorporated, NY, USA). Cells were then incubated for 48 h in the cell culture chamber. To expose the cells, the PMA-containing media was replaced with fresh cell culture media without MWF (control) or supplemented with 0.01% new or used MWF. The cells were then incubated during 24 hours in the cell culture chamber. TNF-α was then analyzed with ELISA using matched capture and detection antibodies (TNF-α ELI-Pair, # 851.570.010). Measurements of chemiluminescence were performed as described for the MPO-analysis and the assay was performed similar to a previously described IL-1β assay [22].

### Statistical methods

Non-parametric Mann-Whitney U test was used as statistical method to calculate significant differences between groups, except for calculations in table 1 where Chi-squared test was used. A *p*≤0.05 was considered statistically significant. All results are given as mean ±standard deviation if not otherwise stated. IBM SPSS Statistics 20 was used as statistical software.

**Table 1:** Demographic characteristics and percent workers that experienced health problems related to work, their work characteristics and the environmental characteristics.

	Percent of workers exposed		
	Directly (n = 102)	Indirectly (n = 169)	Not exposed (n = 24)
**Demographic**			
Men/women	93/7	97/3	88/12
Smokers	13	16	19
**Health problems related to work**			
Irritated, stuffy or runny nose	37**	21	8
Cough	17**	6	4
Dry hands, itching or red skin	11	10	8
Fatigue	12*	22	25
Headache	4	4	8
**Work situation**			
Stimulating work	55	51	29
Too high work rate	20*	10	25
Influence possibilities	34	30	8
**Environmental work problems**			
Dry air	11*	20	38
Odour	20	19	33
Dust and dirt	29	35	57

All health problems and environmental factors were frequently experienced (defined as at least once a week).

p<0.05 and

p<0.01 between directly and indirectly exposed worker.

## Results

### Survey

**Figure 1:**
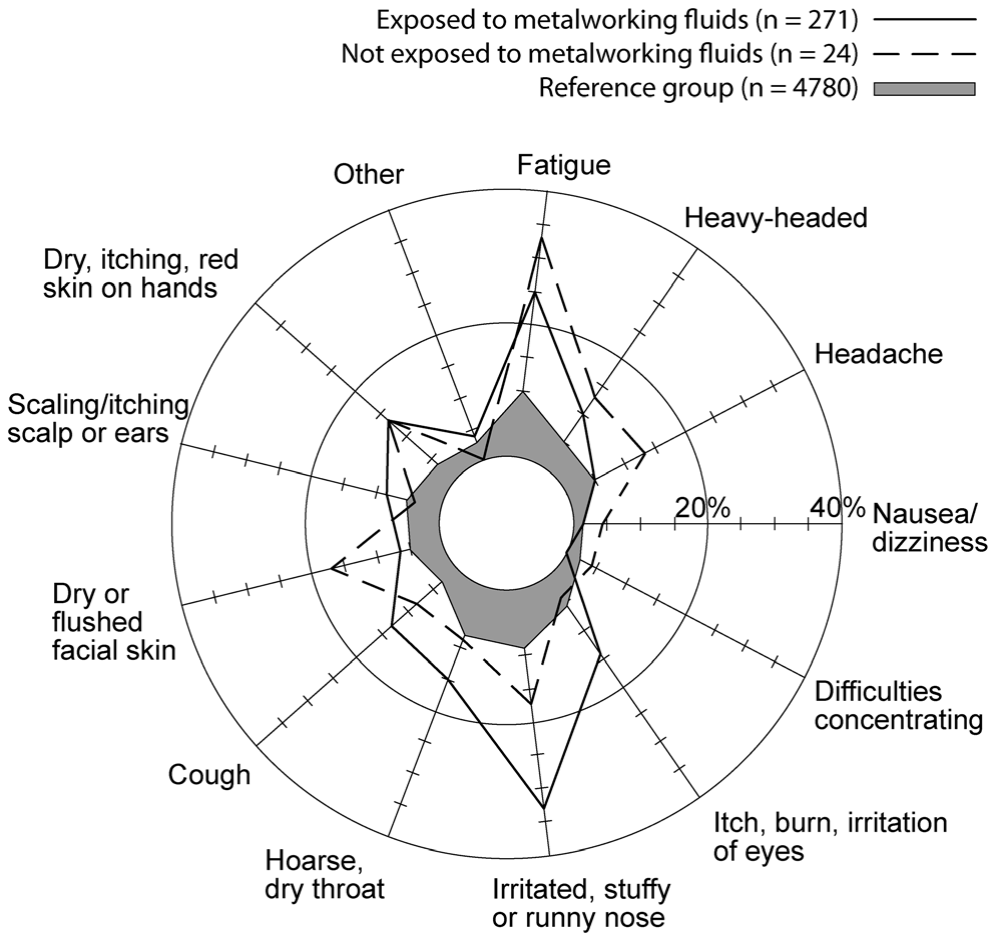
Symptoms reported by metalworkers exposed to metalworking fluid (271 individuals), metal workers not exposed to metalworking fluid (24 individuals) and non-exposed reference population (4780 individuals). The figure shows prevalence of experienced symptoms.

The survey of the metal industry workers (n = 295) showed a high proportion of symptoms compared to a reference group consisting of school and office personnel (n = 4780). Mucous symptoms were the most common problems, which included irritated, stuffy or runny nose, hoarse and dry throat and cough (Figure 1), but also symptoms regarding dry skin on hands or face, eye irritation and fatigue were found. The results showed that workers that operated machines using MWFs (n = 271) had significantly higher prevalence of work-related nasal irritation and coughing compared to unexposed workers (n = 24) at the same factory, 39% compared to 21%, respectively. Among the workers that operated machines with MWF (n = 102) there was a significantly higher fraction with nasal irritation and coughing related to work, 37% and 17%, respectively, compared to those working in the same hall but not handling the MWF (n = 169), 21% and 6%, respectively (Table 1, for more extensive information see Table S1). In the group working in a separate hall and not exposed to MWFs only 8% experienced nasal irritation and 4% had a cough related to work (n = 24). Skin problems were more pronounced among metal workers compared to the reference population, but in contrast to airway problems no statistical differences were found between MWF exposed personnel and unexposed personnel.

### Levels of airborne irritants

Generally, low levels of substances were found (Table 2). All substances were measured close to the work places handling MWF, except for formaldehyde that was measured at several locations, both close and away from the machines. These measurements showed that formaldehyde was evenly distributed in the large processing hall. Both stationary and personal measurements were made, with mean levels of 0.04 mg/m^3^ and 0.1 mg/m^3^, respectively. In contrast, formaldehyde was not detected in the separate mounting hall in which MWF was not used. The levels of morpholine and monoethanolamine were on average 0.53 mg/m^3^ and 0.05 mg/m^3^, respectively. Average levels for both dust and oil mist was 0.46 mg/m^3^ and endotoxin was found in an average concentration of 0.18 ng/m^3^.

**Table 2:** Average indoor air concentrations of MWF generated substances.

Measured substance	(n)	Concentration (mean (min-max))
Morpholine (mg/m^3^)	4	0.53 (0.31–0.7)
Dust (mg/m^3^)	3	0.46 (0.14–0.94)
Oil mist (mg/m^3^)	4	0.46 (0.2–1.2)
Endotoxin (ng/m^3^)	2	0.18 (0.17–0.18)
Monoethanolamine (mg/m^3^)	4	0.05 (0.005–0.16)
Formaldehyde, stationary (mg/m^3^)	18	0.04 (0.02–0.08)
Formaldehyde, personal (mg/m^3^)	4	0.1 (0.02–0.31)

Levels of measured irritants were below the Swedish Work Environment Authority's exposure limit values except oil mist. Of four measured oil mist samples one was above the threshold limit value (1 mg/m^3^) with a concentration of 1.2 mg/m^3^.

### Nasal lavage fluid analysis

**Figure 2:**
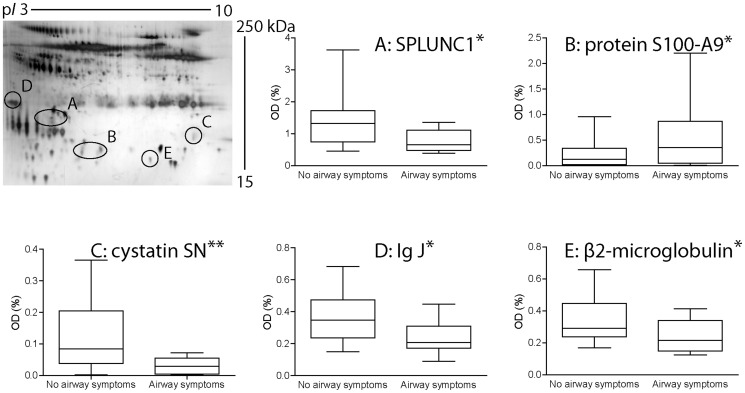
Protein changes in nasal lavage fluid among subjects with airway symptoms compared to subjects without airway symptoms. Proteins were separated with 2-DE, visualized with silver staining and identified by mass spectrometry. Quantitative data (optical density, OD) represent median, interquartile range and max/min-values for 8 subjects with airway symptoms and 20 subjects without airway symptoms. A nasal lavage fluid 2-DE protein pattern is shown in the upper left corner. The proteins were separated according to size from top to bottom (250-15 kDa) and according to isoelectric point from left to right (3–10). *p≤0.05, **p≤0.005 (Mann-Whitney U test).

The results from 2-DE PAGE showed five proteins differently distributed between the two groups that were identified by mass spectrometry (supporting file, Table S2). As illustrated in figure 2, SPLUNC1, cystatin SN, Ig J and β2-microglobulin was 30–60% less abundant while protein S100-A9 was about 3-fold more abundant in nasal lavage fluid from the subjects with airway symptoms compared to the subjects without airway symptoms. A significant decreased expression of SPLUNC1 and increased expression of S100-A9 was confirmed by western blot analysis (supporting file, Figure S1) In addition, MPO levels were slightly higher in the group with airway symptoms, 8.3±5.8 ng/ml, compared to the group without airway symptoms, 6.7±4.1 ng/ml. There were no significant correlations between MPO levels and the levels of SPLUNC1, S100-A9, Cystatin SN, IgJ or β2-microglobolin. The level of IL-1β was significantly higher in subjects with airway symptoms (16.2±4.2 pg/ml) compared to subjects without airway symptoms (10.6±6.2 pg/ml), (p = 0.043). Total protein concentration was not statistically different between subjects with airway symptoms (8 workers) and subjects without airway symptoms (5 workers and 15 controls); 101±35 µg/ml and 140±65 µg/ml, respectively. No differences were found between workers without airway symptoms and controls.

### NO measurement

All subjects had NO levels below 30 ppb. NO levels in the subjects with airway symptoms was somewhat higher compared to the subjects without symptoms, 17±6.9 ppb and 14±6.3 ppb, respectively, but did not reach statistical significance.

### MWF-mediated cellular TNF-α production *in vitro*

**Figure 3:**
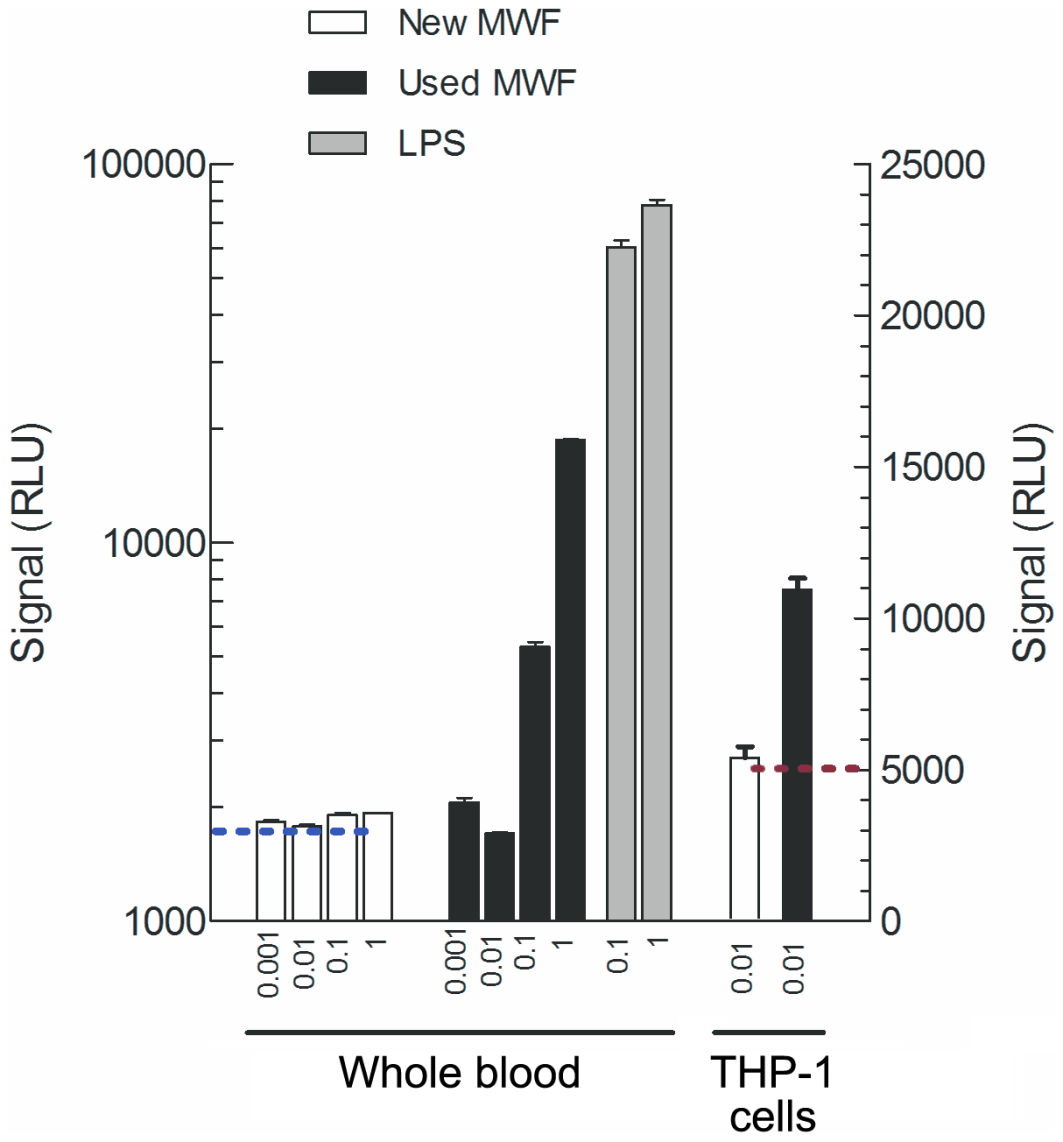
TNF-α production from whole blood or THP-1 cells after stimulation with new or used metalworking fluid (MWF) measured with ELISA and expressed as relative luminescence units (RLU). Whole blood sample was incubated during 4% to 1% of new and used MWF or LPS (positive control) diluted in 0.9% NaCl. THP-1 cells were incubated during 24 hours with 0.01% new or used MWF in culture medium. For details see Materials and method. Each bar represents mean ± SD of two experiments.

The ability of used and new MWF to stimulate TNF-α production was compared using a whole blood assay. As shown in Figure 3, used MWF was about 3-fold (0.1% MWF) and 10-fold (1% MWF) more potent to induce TNF-α formation than new, not used, MWF. Similar difference in response was also found in THP-1 monocytes/macrophages.

### Effect of preventive actions

The follow-up questionnaires performed before and after improvement of the ventilation of the machines, showed a reduction in the prevalence of airway symptoms after the measures were taken. Before the rebuilding of ventilation, 43% of all MWF exposed personnel (n = 221) experienced almost daily discomfort from the airways. Of those exposed directly (n = 87) and those exposed indirectly (n = 134) to MWF; 48% and 39%, respectively, experienced almost daily discomfort from airways. Six months later, when the rebuilding of the ventilation systems were completed, the prevalence of airway problems was reduced from 43% to 21% in the MWF exposed personnel (n = 219). Among the personnel exposed directly to MWF (n = 93) 30% experienced airway symptoms and among the personnel exposed indirectly to MWF (n = 126) only 13% had upper respiratory problems. In addition, the formaldehyde levels were reduced by about 50% and the levels ranged from 0.02 mg/m^3^ to not detectable in the measurements performed after improvement of the ventilation.

## Discussion

In the current study we demonstrate high prevalence of upper airway mucosal symptoms among metal industry workers exposed to MWF. Many of the symptoms, e.g. nasal blockage, runny nose and sore throat, are similar to those found in acute rhinosinusitis and allergic rhinitis [23]. Although the symptoms were found especially in the subjects handling the MWF containing machineries they were also reported from those indirectly exposed to MWF. For example, nasal irritation related to work was found in 37% of the workers directly exposed to MWF, in 21% of the subjects working in the same hall but not handling the machines using MWF and in 8% of those that worked in a separate building not exposed to MWF. Similar results has been shown by Greaves *et al*, where they found about a two-fold higher risk for upper airway symptoms in subjects working with synthetic MWF compared to assembly workers [3]. In contrast, skin problems related to work were not connected to MWF exposure, about 10% prevalence in all three groups. According to the survey, the airway problems were not connected to unpleasant psychosocial working conditions for the MWF exposed workers. Instead they reported more often that they had a stimulating work (50–55%) and possibilities to influence their work situation (30–35%) than the workers not exposed to MWF (29% and 8%, respectively). In addition, discomfort from common indoor air factors such as dry air, odour and dust was less frequent among those in the metal working hall compared to those working in the mounting hall and not exposed to MWF (Table 1).

NLF contains a large number of proteins that altogether comprise a potential source of detecting and characterizing biochemical alterations associated with airway diseases (supporting files, Table S3 and Figure S2). Our proteomic analysis revealed five significant effects in the protein distribution pattern of NLF from the workers with airway symptoms; more of protein S100-A9 (calgranulin B) and less of SPLUNC1, cystatin SN, Ig J and β2-microglobulin, all of which are involved in the immune system. Protein S100-A9 is highly expressed in the cytoplasm of neutrophils and is found in high levels in extracellular fluids during inflammatory diseases, such as chronic inflammatory bowel disease and rheumatoid arthritis, usually in complex with the related protein S100-A8 (calprotectin) [24]. Under these conditions they may also be produced by epithelial cells [25]. The proteins are important for the mucosal defence against microbes and are a part of the endogenous danger associated molecular pattern (DAMP) to boost the innate immunity responses [26]–[27]. The function of the proteins is not fully understood and appears to be complex as both pro- and anti-inflammatory effects have been described [28]–[29]. It is possible that this is related to different functions of the two S100 proteins as, in mice, S100-A8 acts as an activator of Toll-like receptor 4 promoting endotoxin induced inflammation while S100-A9 appear to have a more regulatory role [30]. Notably, the levels of S100-A8 were not significantly altered in the subjects with symptoms although we recently have found decreased levels of S100-A8, but unaltered levels of S100-A9, in swimming pool personnel with similar upper airway symptoms as the MWF exposed workers [31]. This may indicate that a disturbed balance between the two S100 proteins is a common feature of upper respiratory mucosal inflammation. SPLUNC1 is an endotoxin-binding protein (also known as BPI fold-containing family A member 1, BPIFA1) highly expressed in the upper airways and was initially discovered as a possible biomarker for environmental-induced airway irritation [32]. In line, lower levels of SPLUNC1 have been found in epoxy workers exposed to reactive chemicals and smokers [33]–[34]. Further studies have shown that SPLUNC1 is part of the innate immune system and prevents bacterial growth and biofilm formation, partly by acting as an upper airway surfactant protein [35]–[38]. SPLUNC1 is an abundant protein in the upper airways and its importance has further been highlighted by a recent study showing that SPLUNC1 is needed to attenuate long-term airway inflammation in mice exposed to carbon nanotubes [39]. Cystatin SN belongs to the cystatin super family, which are highly expressed in saliva and inhibits cystein peptidases [40]. The protein therefore acts as an anti-virulence factor in the airways and protects inflamed tissue against excessive injury [41]–[42]. Notably, cystatin S, an other member of the cystatin family expressed in the upper airways, is affected in smokers [20]. Finally, both Ig J and β2-microglobulin are part of the adaptive immune response as they are necessary for the formation of Ig M and Ig A, respectively, which are involved in antigen presentation [43]–[44]. Taken together, these results imply effects on the immune system among the workers exposed to MWF that may contribute to the upper airway inflammation. However, it is important to point out that the protein findings are based on a relatively small number of subjects and more extended studies are needed to verify the effects. In addition, the mechanisms behind the effects can only be speculated upon. Both cystatins and SPLUNC1 have been shown to be targets of neutrohil elastase activity [45]–[46]. Therefore, it is possible that the decreased levels of the proteins are caused by increased degradation mediated by activated neutrophils, which would be in line with release of S100-A9. However, although the levels of MPO, an enzyme primarily found in azurophilic granules and used as a neutrophil marker, was slightly increased in the workers with symptoms there were no significant difference from the subjects without symptoms. Furthermore, there were no correlations between the MPO levels and the levels of SPLUNC1, cystatin SN, S100-A9, Ig J and β2-microglobulin. So, it is possible to speculate that exposure to MWF containing a mixture of reactive and pro-inflammatory agents may induce a toxic response in the upper airway cell lining, resulting in decreased formation of normally expressed immune defense proteins as well as release of pre-formed immune proteins without signal peptides, such as DAMPs.

Several potential airway irritants; formaldehyde, ethanolamine, morpholine and oil mist, from the MWF were detected in the metal working hall. However, they were all measured in low concentrations that one by one are probably not enough to cause airway symptoms. Instead, it is possible that together they create a synergistic cocktail effect. Formaldehyde is a well-known upper airway irritant and released as a bacteriostatic agent in the MWF used at the factory. In contrast, it was not detected in the separate mounting hall that did not include metal working. As a marker of MWF exposure, formaldehyde was found in approximately the same concentrations throughout the hall implicating why not only workers directly handling the cutting tools were affected. Besides, low levels of endotoxin were detected, which indicates possible bacterial contamination of the MWF that may have contributed to the effects. Interestingly, our *in vitro* test showed that a sample of used MWF taken directly from a cutting machine at the factory had considerably higher inflammatory promoting effect than unused MWF. Whether this is caused by higher amounts of anti-bacterial formaldehyde, microbial growth and generation of pro-inflammatory products such as endotoxin or enrichment of particulate matter is not known. However, it does illustrate that the MWF used in the production, over time, probably becomes more likely to cause airway inflammation. It has previously been suggested that endotoxin contamination of MWF is the causative agent responsible for inducing an inflammatory response in airways [12], [47]. In relation to our protein findings, it has recently been shown that recombinant SPLUNC1 reduces TNF-α release from activated macrophages [39] and may be important to prevent dehydration of the airway mucosa [48]. It is therefore possible to speculate that the lower amounts of SPLUNC1 found in the workers with airway symptoms may be one contributing mechanism for the upper airway inflammation in the subjects.

The studied metal factory had a history of skin problems among employees handling MWF. The company therefore decided to use water-based instead of oil based MWF and the cutting machines were equipped with oil mist separators to improve the air quality. However, these types of separators only remove oil mist mechanically without preventing gases and aerosols generated from the water-based MWF to spread to the work environment. As shown by our survey the health issues then turned from skin problems to a high prevalence of upper airway symptoms, also among those working in the same hall but not handling the machines. Most likely, the problem was aggravated by the fact that the air from the separators was recycled back into the factory hall. When this was corrected and the air from oil mist separators, together with the regular ventilation was instead redirected out of the building, the levels of formaldehyde, monitored as marker of MWF exposure, decreased. More importantly, the number of workers with airway symptoms was reduced considerably, especially in the group indirectly exposed to MWF. In the group handling the MWF-containing machines a significant portion still experienced airway symptoms, implicating the need of further improvements of the work conditions. This could include improving working routines, e.g. lowering the use of compressed air as a cleaning technique to reduce spread of MWF aerosol, more frequent monitoring bacterial growth in MWF and more often substitution of used MWF, as well as further enclosure of the machines to ensure efficient ventilation [8].

In summary, this study confirms that airway symptoms among metal workers exposed to MWF is a common problem. We have, despite low levels of MWF generated substances, shown widespread airway symptoms among metal workers in connection with MWF exposure and found effects on airway immune proteins in subjects with symptoms. Furthermore, we have shown how preventive action can improve work environment and health status. Future studies should aim to further improve the working conditions for workers handling MWF and to clarify how the airway inflammation is connected to specific MWF components and to the identified biological effect or markers.

## Supporting Information

Figure S1 **Representative Western blots of expression level of SPLUNC1 and S100-A9.** Lane A and B are representative examples of SPLUNC1 and S100-A9 expression level in nasal lavage fluid from subjects without airway symptoms, lane C and D are from subjects with airway symptoms. The quantification data (Optical Density, OD), from subjects with no airway symptoms (n = 20) and with airway symptoms (n = 8) are shown in histogram.
(TIF)

Figure S2 **A typical 2-DE gel map of nasal lavage fluid. Separated proteins were detected by silver staining.** Annotations correspond to spot numbers in Table S3.
(TIF)

Table S1 **Extended version of** **table 1** **.** Work related health problems and environmental factors of 295 workers at the metal industry. Comparison of workers exposed directly to metalworking fluid (n = 102), workers exposed indirectly to metalworking fluid (n = 169) and workers not exposed to metalworking fluids (n = 24). All health problems and environmental factors are frequently experienced, i.e. at least once a week, except for asthma, hay fever, eczema and allergy in family.
(DOCX)

Table S2 **Mass spectrometric data over the five proteins that showed significant differences between subjects with and without airway symptoms.** Protein spots were excised from the gel, destained and in-gel digested with trypsin. The tryptic peptides were analyzed by MALDI-TOF MS.
(DOCX)

Table S3 **Identified proteins in nasal lavage fluid.** The numbers referred to the spot number in Figure S2.
(DOCX)

## References

[1] OSHA (1999) U.S. Department of Labour: “Metalworking Fluids: Safety and Health Best Practices Manual”. Available at http://www.osha.gov/SLTC/metalworkingfluids/metalworkingfluids_manual.html. Accessed 17 April 2013.

[2] Rosenman KD (2009) Asthma, hypersensitivity pneumonitis and other respiratory diseases caused by metalworking fluids. Curr Opin Allergy Clin Immunol 9: 97–102. 19307882 10.1097/ACI.0b013e3283229f96

[3] Greaves IA, Eisen EA, Smith TJ, Pothier LJ, Kriebel D, et al (1997) Respiratory health of automobile workers exposed to metal-working fluid aerosols: respiratory symptoms. Am J Ind Med 32: 450–9. 9327068 10.1002/(sici)1097-0274(199711)32:5<450::aid-ajim4>3.0.co;2-w

[4] Jaakkola MS, Suuronen K, Luukkonen R, Järvelä M, Tuomi T, et al (2009) Respiratory symptoms and conditions related to occupational exposures in machine shops. Scand J Work Environ Health 35: 64–73. 19190832 10.5271/sjweh.1299

[5] Gruvberger B, Isaksson M, Frick M, Pontén A, Bruze M (2003) Occupational dermatoses in a metalworking plant. Contact Dermatitis 48: 80–6. 12694210 10.1034/j.1600-0536.2003.480205.x

[6] Graff P, Elmsjo L, Bjorkander J, Flodin U (2008) Occupational rhinitis caused by tolyltriazole in metalworking fluids. Scand J Work Environ Health 34: 403–4. 18853069 10.5271/sjweh.1276

[7] Chen MR, Tsai PJ, Chang CC, Shihc TS, Lee WJ, et al (2007) Particle size distributions of oil mists in workplace atmospheres and their exposure concentrations to workers in a fastener manufacturing industry. J Hazard Mater 146: 393–8. 17222970 10.1016/j.jhazmat.2006.12.036

[8] Lillienberg L, Burdorf A, Mathiasson L, et al (2008) Exposure to metalworking fluid aerosols and determinants of exposure. Ann Occup Hyg 52: 597–605. 18664515 10.1093/annhyg/men043

[9] Suuronen K, Henriks-Eckerman ML, Riala R, Tuomi T (2008) Respiratory exposure to components of water-miscible metalworking fluids. Ann Occup Hyg 52: 607–14. 18678881 10.1093/annhyg/men048

[10] Sollenberg J, Ståhlbom B (1998) Emission of morpholine from metalworking fluids. Occup Hyg 4 (3–6) 281–8.

[11] Henriks-Eckerman ML, Suuronen K, Jolanki R, Riala R, Tuomi T (2007) Determination of occupational exposure to alkanolamines in metal-working fluids. Ann Occup Hyg 51: 153–60. 17189280 10.1093/annhyg/mel079

[12] Gordon T (2004) Metalworking fluid–the toxicity of a complex mixture. J Toxicol Environ Health A 67: 209–219. 14681076 10.1080/15287390490266864

[13] Marchand G, Lavoie J, Racine L, Lacombe N, Cloutier Y, et al (2010) Evaluation of bacterial contamination and control methods in soluble metalworking fluids. J Occup Environ Hyg 7: 358–66. 20379898 10.1080/15459621003741631

[14] Trafny EA (2013) Microorganisms in metalworking fluids: Current issues in research and management. Int J Occup Med Environ Health 26: 4–15. 23526197 10.2478/S13382-013-0075-5

[15] Murat JB, Grenouillet F, Reboux G, Penven E, Batchili A, et al (2012) Factors Influencing the Microbial Composition of Metalworking Fluids and Potential Implications for Machine Operator's Lung. Appl Environ Microbiol 10.1128/AEM.06230-11 PMC3255630 22057869

[16] Andersson K (1998) Epidemiological Approach to Indoor Air Problems. Indoor air 8: 32–9.

[17] Taylor DR, Pijnenburg MW, Smith AD, Jongste JCD (2006) Exhaled nitric oxide measurements: clinical application and interpretation. Thorax 61: 817–27. 16936238 10.1136/thx.2005.056093 PMC2117092

[18] Bradford MM (1976) A rapid and sensitive method for the quantitation of microgram quantities of protein utilizing the principle of protein-dye binding. Anal Biochem 72: 248–54. 942051 10.1016/0003-2697(76)90527-3

[19] Ghafouri B, Karlsson H, Mortstedt H, Lewander A, Tagesson C, et al (2007) 2,5-Dihydroxybenzoic acid instead of alpha-cyano-4-hydroxycinnamic acid as matrix in matrix-assisted laser desorption/ionization time-of-flight mass spectrometry for analyses of in-gel digests of silver-stained proteins. Anal Biochem 371: 121–3. 17723224 10.1016/j.ab.2007.07.002

[20] Ghafouri B, Stahlbom B, Tagesson C, Lindahl M (2002) Newly identified proteins in human nasal lavage fluid from non-smokers and smokers using two-dimensional gel electrophoresis and peptide mass fingerprinting. Proteomics 2: 112–120. 11788998

[21] Chang PY, Wu TL, Hung CC, Tsao KC, Sun CF, et al (2006) Development of an ELISA for myeloperoxidase on microplate: normal reference values and effect of temperature on specimen preparation. Clin Chim Acta 373: 158–63. 16815352 10.1016/j.cca.2006.05.030

[22] Jayawardena U, Tollemark L, Tagesson C, Leanderson P (2009) Pyrogenic effect of respirable road dust particles. J Phys Conf Ser 151.

[23] Fokkens WJ, Lund VJ, Mullol J, Bachert C, Alobid I, et al (2012) European Position Paper on Rhinosinusitis and Nasal Polyps 2012. Rhinol Suppl: 3 preceding table of contents, 1–298. 22764607

[24] Foell D, Frosch M, Sorg C, Roth J (2004) Phagocyte-specific calcium-binding S100 proteins as clinical laboratory markers of inflammation. Clin Chim Acta 344: 37–51. 15149869 10.1016/j.cccn.2004.02.023

[25] Frosch M, Metze D, Foell D, Vogl T, Sorg C, et al (2005) Early activation of cutaneous vessels and epithelial cells is characteristic of acute systemic onset juvenile idiopathic arthritis. Exp Dermatol 14: 259–265. 15810883 10.1111/j.0906-6705.2005.00271.x

[26] Champaiboon C, Sappington KJ, Guenther BD, Ross KF, Herzberg MC (2009) Calprotectin S100A9 calcium-binding loops I and II are essential for keratinocyte resistance to bacterial invasion. J Biol Chem 284: 7078–90. 19122197 10.1074/jbc.M806605200 PMC2652321

[27] Foell D, Wittkowski H, Vogl T, Roth J (2007) S100 proteins expressed in phagocytes: a novel group of damage-associated molecular pattern molecules. J Leukoc Biol 81: 28–37. 16943388 10.1189/jlb.0306170

[28] Halayko AJ, Ghavami S (2009) S100A8/A9: a mediator of severe asthma pathogenesis and morbidity? Can J Physiol Pharmacol 87: 743–55. 19898558 10.1139/Y09-054

[29] Goyette J, Geczy CL (2010) Inflammation-associated S100 proteins: new mechanisms that regulate function. Amino Acids 10.1007/s00726-010-0528-0 20213444

[30] Vogl T, Tenbrock K, Ludwig S, Leukert N, Ehrhardt C, et al (2007) Mrp8 and Mrp14 are endogenous activators of Toll-like receptor 4, promoting lethal, endotoxin-induced shock. Nat Med 13: 1042–9. 17767165 10.1038/nm1638

[31] Fornander L, Ghafouri B, Lindahl M, Graff P (2013) Airway irritation among indoor swimming pool personnel: trichloramine exposure, exhaled NO and protein profiling of nasal lavage fluids. Int Arch Occup Environ Health 86: 571–80. 22729567 10.1007/s00420-012-0790-4

[32] Lindahl M, Stahlbom B, Tagesson C (2001) Identification of a new potential airway irritation marker, palate lung nasal epithelial clone protein, in human nasal lavage fluid with two-dimensional electrophoresis and matrix-assisted laser desorption/ionization-time of flight. Electrophoresis 22: 1795–800. 11425234 10.1002/1522-2683(200105)22:9<1795::AID-ELPS1795>3.0.CO;2-J

[33] Ghafouri B, Stahlbom B, Tagesson C, Lindahl M (2002) Newly identified proteins in human nasal lavage fluid from non-smokers and smokers using two-dimensional gel electrophoresis and peptide mass fingerprinting. Proteomics 2: 112–20. 11788998

[34] Ghafouri B, Kihlstrom E, Tagesson C, Lindahl M (2004) PLUNC in human nasal lavage fluid: multiple isoforms that bind to lipopolysaccharide. Biochim Biophys Acta 1699: 57–63. 15158712 10.1016/j.bbapap.2004.01.001

[35] Canny G, Levy O (2008) Bactericidal/permeability-increasing protein (BPI) and BPI homologs at mucosal sites. Trends Immunol 29: 541–7. 18838299 10.1016/j.it.2008.07.012

[36] Bingle CD, Craven CJ (2004) Meet the relatives: a family of BPI- and LBP-related proteins. Trends Immunol 25: 53–5. 15106612 10.1016/j.it.2003.11.007

[37] Lukinskiene L, Liu Y, Reynolds SD, Steele C, Stripp BR, et al (2011) Antimicrobial activity of PLUNC protects against Pseudomonas aeruginosa infection. J Immunol 187: 382–90. 21632717 10.4049/jimmunol.1001769 PMC3119743

[38] Gakhar L, Bartlett JA, Penterman J, Mizrachi D, Singh PK, et al (2010) PLUNC is a novel airway surfactant protein with anti-biofilm activity. PLoS One 5: e9098. 20161732 10.1371/journal.pone.0009098 PMC2817724

[39] Di YP, Tkach AV, Yanamala N, Stanley S, Gao S, et al (2013) Dual Acute Pro-Inflammatory and Anti-Fibrotic Pulmonary Effects of SPLUNC1 After Exposure to Carbon Nanotubes. Am J Respir Cell Mol Biol 10.1165/rcmb.2012-0435OC PMC3931096 23721177

[40] Isemura S, Saitoh E, Sanada K (1986) Characterization of a new cysteine proteinase inhibitor of human saliva, cystatin SN, which is immunologically related to cystatin S. FEBS Lett. 198: 145–9. 10.1016/0014-5793(86)81201-7 3514272

[41] Fietta A, Bardoni A, Salvini R, Passadore I, Morosini M, et al (2006) Analysis of bronchoalveolar lavage fluid proteome from systemic sclerosis patients with or without functional, clinical and radiological signs of lung fibrosis. Arthritis Res Ther 8: R160. 17044913 10.1186/ar2067 PMC1794502

[42] Turk V, Stoka V, Turk D (2008) Cystatins: biochemical and structural properties, and medical relevance. Front Biosci 13: 5406–20. 18508595 10.2741/3089

[43] Frutiger S, Hughes GJ, Paquet N, Luethy R, Jaton JC (1992) Disulfide bond assignment in human J chain and its covalent pairing with immunoglobulin M. Biochemistry. 31: 12643–7. 10.1021/bi00165a014 1472500

[44] Rosano C, Zuccotti S, Bolognesi M (2005) The three-dimensional structure of beta2 microglobulin: results from X-ray crystallography. Biochim Biophys Acta 1753: 85–91. 16084780 10.1016/j.bbapap.2005.07.010

[45] Abrahamson M, Wikstrom M, Potempa J, Renvert S, Hall A (1997) Modification of cystatin C activity by bacterial proteinases and neutrophil elastase in periodontitis. Mol Pathol 50: 291–297. 9536278 10.1136/mp.50.6.291 PMC379662

[46] Jiang D, Wenzel SE, Wu Q, Bowler RP, Schnell C, et al (2013) Human neutrophil elastase degrades SPLUNC1 and impairs airway epithelial defense against bacteria. PLoS One 8: e64689. 23741370 10.1371/journal.pone.0064689 PMC3669426

[47] DeLorme MP, Gao X, Doyon-Reale N, Barraclough-Mitchell H, Bassett DJ (2003) Inflammatory effects of inhaled endotoxin-contaminated metal working fluid aerosols in rats. J Toxicol Environ Health A 66: 7–24. 12587288 10.1080/15287390306458

[48] Garland AL, Walton WG, Coakley RD, Tan CD, Gilmore RC, et al (2013) Molecular basis for pH-dependent mucosal dehydration in cystic fibrosis airways. Proc Natl Acad Sci U S A 110: 15973–15978. 24043776 10.1073/pnas.1311999110 PMC3791714

