# Supplementary figures and images for: Airway Symptoms and Biological Markers in Nasal Lavage Fluid in Subjects Exposed to Metalworking Fluids
Source: PLoS One. 2013 Dec 31;8(12):e83089. doi: 10.1371/journal.pone.0083089 (PMC3877012; doi:10.1371/journal.pone.0083089)

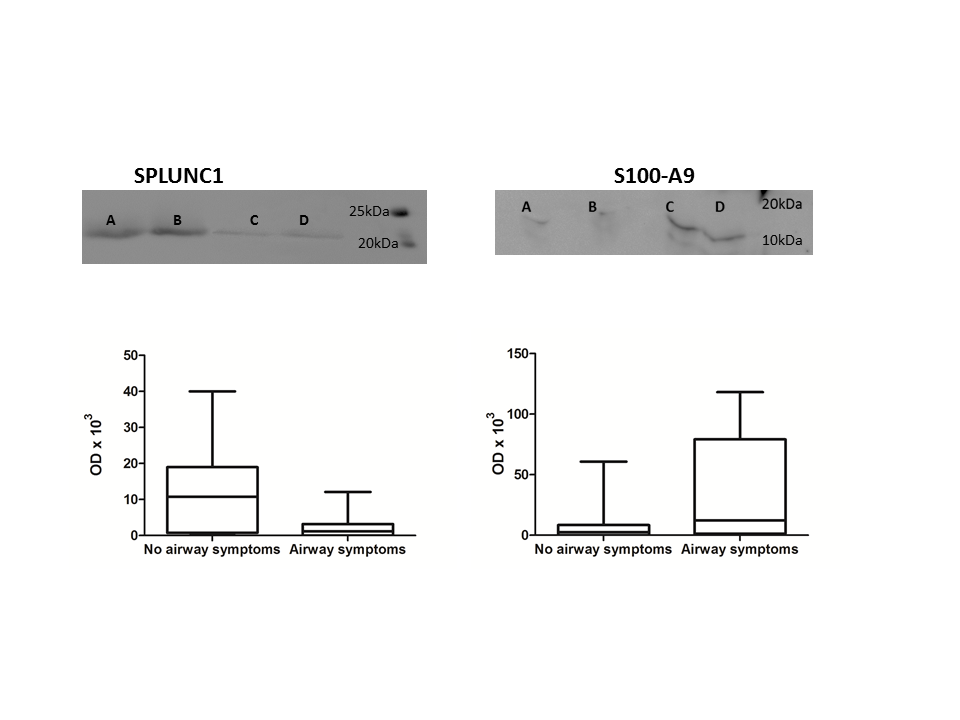

Supplement: Figure S1 — Representative Western blots of expression level of SPLUNC1 and S100-A9. Lane A and B are representative examples of SPLUNC1 and S100-A9 expression level in nasal lavage fluid from subjects without airway symptoms, lane C and D are from subjects with airway symptoms. The quantification data (Optical Density, OD), from subjects with no airway symptoms (n = 20) and with airway symptoms (n = 8) are shown in histogram. (TIF) [file pone.0083089.s001.tif]

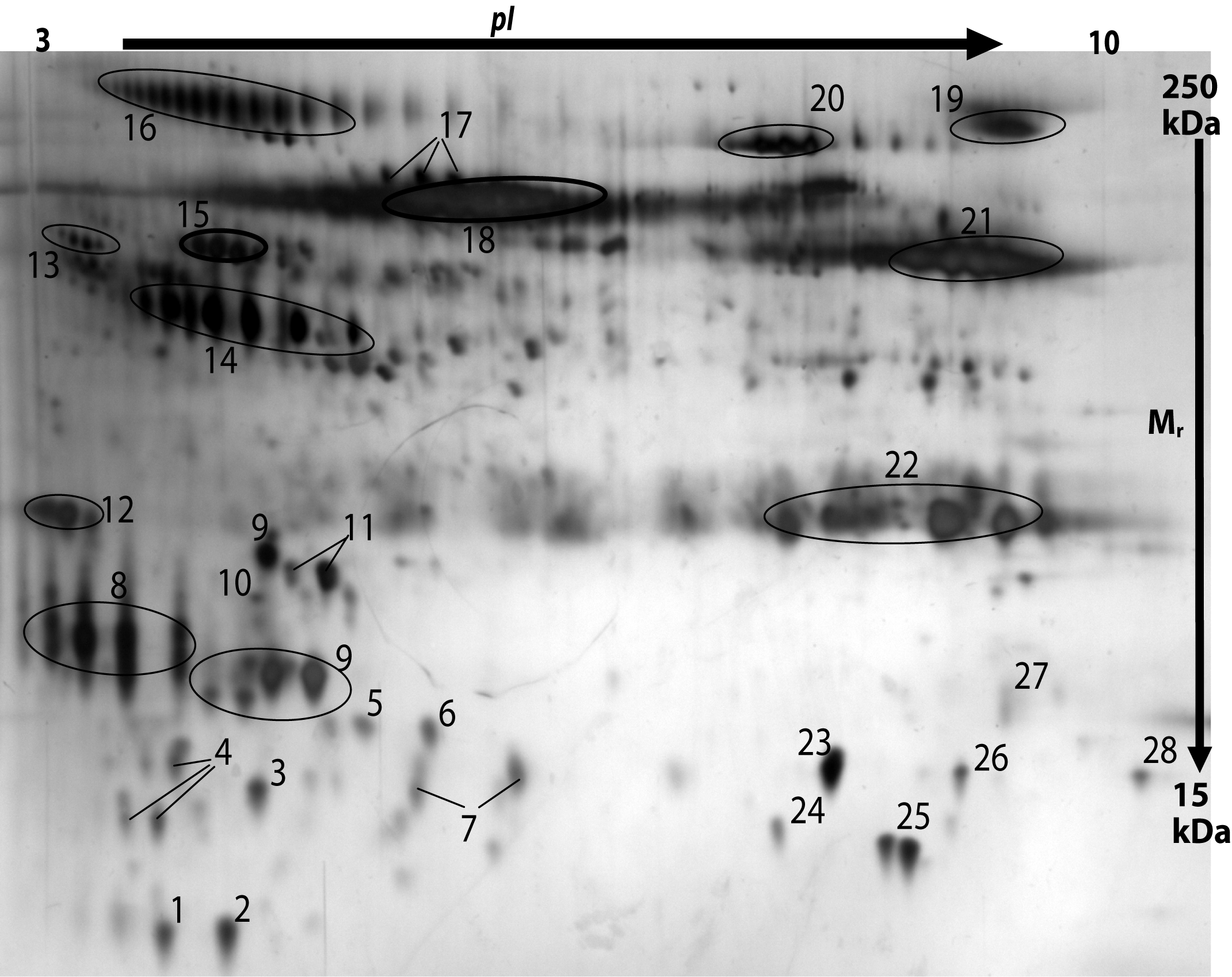

Supplement: Figure S2 — A typical 2-DE gel map of nasal lavage fluid. Separated proteins were detected by silver staining. Annotations correspond to spot numbers in Table S3. (TIF) [file pone.0083089.s002.tif]
