# Supplementary material for: Airway Symptoms and Biological Markers in Nasal Lavage Fluid in Subjects Exposed to Metalworking Fluids
Source: PLoS One. 2013 Dec 31;8(12):e83089. doi: 10.1371/journal.pone.0083089 (PMC3877012; doi:10.1371/journal.pone.0083089)
Supplement: Table S1 — Extended version of table 1 . Work related health problems and environmental factors of 295 workers at the metal industry. Comparison of workers exposed directly to metalworking fluid (n = 102), workers exposed indirectly to metalworking fluid (n = 169) and workers not exposed to metalworking fluids (n = 24). All health problems and environmental factors are frequently experienced, i.e. at least once a week, except for asthma, hay fever, eczema and allergy in family. (DOCX) [file pone.0083089.s003.docx]

Table S1 Extended version of table 1. Work related health problems and environmental factors of 295 workers at the metal industry. Comparison of workers exposed directly to metalworking fluid (n = 102), workers exposed indirectly to metalworking fluid (n = 169) and workers not exposed to metalworking fluids (n = 24). All health problems and environmental factors are frequently experienced, i.e. at least once a week, except for asthma, hay fever, eczema and allergy in family.

|  | Exposed to metalworking fluids | | Not exposed to metalworking fluids | | |
| --- | --- | --- | --- | --- | --- |
|  | Exposed directly  (%) | Exposed indirectly (%) | (%) | | |
|  |  |  |  | | |
| Demographic |  |  |  | | |
| Men/women | 93/7 | 97/3 | 88/12 | | |
| Smokers | 13 | 16 | 19 | | |
|  |  |  |  | | |
| Present/former disease |  |  |  | | |
| Asthma* | 11 | 15 | 17 | | |
| Hay fever* | 25 | 23 | 25 | | |
| Eczema* | 25 | 25 | 17 | | |
| Allergy in family | 31 | 31 | 46 | | |
|  |  |  |  | | |
| Health problems (total answer rate/answer due to work) | | | |  |  |
| Fatigue | 19/12 | 30/22 | 33/25 | |  |
| Feeling heavy-headed | 8/7 | 11/10 | 13/8 | |  |
| Headache | 4/4 | 5/4 | 13/8 | |  |
| Nausea/dizziness | 0/0 | 1/1 | 4/0 | |  |
| Difficulties concentrating | 0/0 | 1/1 | 4/0 | |  |
| Itching, burning or irritation of the eyes | 13/13 | 15/12 | 4/4 | |  |
| Irritated, stuffy or runny nose | 39/37 | 29/21 | 17/8 | |  |
| Hoarse, dry throat | 16/14 | 14/10 | 8/4 | |  |
| Cough | 17/17 | 10/6 | 8/4 | |  |
| Dry or flushed facial skin | 5/4 | 9/7 | 17/13 | |  |
| Scaling/itching scalp or ears | 10/6 | 8/5 | 4/0 | |  |
| Hands dry, itching, red skin | 13/11 | 12/10 | 13/8 | |  |
| Other | 4/3 | 4/2 | 0/0 | |  |
|  |  |  |  | |  |
| Environmental work problems | |  |  | |  |
| Draught | 14 | 34 | 46 | |  |
| Room temp. too high | 5 | 11 | 21 | |  |
| Varying room temp. | 14 | 25 | 33 | |  |
| Room temp. too low | 8 | 17 | 25 | |  |
| Stuffy “bad” air | 24 | 28 | 38 | |  |
| Dry air | 11 | 20 | 38 | |  |
| Unpleasant odour | 20 | 19 | 33 | |  |
| Static electricity, often causing shocks | 1 | 2 | 4 | |  |
| Passive smoking | 0 | 4 | 17 | |  |
| Noise | 45 | 53 | 50 | |  |
| Light that is dim or cause glare and/or reflections | 21 | 28 | 67 | |  |
| Dust and dirt | 29 | 35 | 57 | |  |
|  |  |  |  | |  |
| Work situation |  |  |  | |  |
| Stimulating work | 55 | 51 | 29 | |  |
| Too high work rate | 20 | 10 | 25 | |  |
| Influence possibilities | 34 | 30 | 8 | |  |
| Help from colleagues | 81 | 73 | 63 | |  |

*have or have had
