# Supplementary material for: Airway Symptoms and Biological Markers in Nasal Lavage Fluid in Subjects Exposed to Metalworking Fluids
Source: PLoS One. 2013 Dec 31;8(12):e83089. doi: 10.1371/journal.pone.0083089 (PMC3877012; doi:10.1371/journal.pone.0083089)
Supplement: Table S2 — Mass spectrometric data over the five proteins that showed significant differences between subjects with and without airway symptoms. Protein spots were excised from the gel, destained and in-gel digested with trypsin. The tryptic peptides were analyzed by MALDI-TOF MS. (DOCX) [file pone.0083089.s004.docx]

Table S2. Mass spectrometric data over the five proteins that showed significant differences between subjects with and without airway symptoms.

| Symbol | Protein | Accession No. | p*I*/MW (Da) | Sequence coverage (%) | Submitted peaks | Theoretical peaks |
| --- | --- | --- | --- | --- | --- | --- |
| A | SPLUNC1 (BPI fold-containing family A member 1) | Q9NP55 | 5.4/26713 | 35.9 | 1116.6778 1213.7384 1480.9198 1595.9192 2074.1318 3604.5133 | 1116.7027 1213.7151 1480.8846 1595.9367 2074.0927 2604.4582 |
| B | Protein S100-A9 | P06702 | 5.7/13242 | 56.1 | 877.4673 971.4453 1341.5208 2114.0125 2191.8445 | 877.4778 971.4945 1341.5960 2114.0070 2191.9574 |
| C | Cystatin SN | P01037 | 6.7/16388 | 50.4 | 731.2877 1292.6589 1897.9504 1914.9743 2073.0617 2142.0041 2142.0041 | 731.2995 1292.6634 1897.9079 1914.9345 2073.0400 2141.9920 2142.0727 |
| D | Immunoglobulin J chain | P01591 | 5.1/18099 | 19.5 | 938.5732 1228.8070 1389.7084 | 938.5782 1228.6168 1389.6128 |
| E | Β2-microglobulin | P61769 | 6.1/13715 | 40.3 | 813.3099 1122.6216 1148.5678 2554.2915 | 813.3196 1122.6266 1148.5582 2554.2283 |
