# Supplementary material for: Airway Symptoms and Biological Markers in Nasal Lavage Fluid in Subjects Exposed to Metalworking Fluids
Source: PLoS One. 2013 Dec 31;8(12):e83089. doi: 10.1371/journal.pone.0083089 (PMC3877012; doi:10.1371/journal.pone.0083089)
Supplement: Table S3 — Identified proteins in nasal lavage fluid. The numbers referred to the spot number in Figure S2. (DOCX) [file pone.0083089.s005.docx]

Table S3. Identified proteins in nasal lavage fluid. The numbers referred to the spot number in Figure S1.

| **Spot**  **no.** | **Accession no SWISS-PROT** | **Protein name** | ***Mw* (kDa*)/pI*** |
| --- | --- | --- | --- |
| 1 | P11684 | CC16 | 6.5/4.8 |
| 2 | O75556 | Lipophilin C | 5.5 /5.3 |
| 3 | P02766 | Transthyretin | 14/5.6 |
| 4 | P01036 | Cystatin S | 13.5/4.9 |
| 5 | P08118 | IgBF | 13.5/5.6 |
| 6 | P02766 | Transthyretin | 14/5.6 |
| 7 | P06702 | Calgranulin B | 13.2 /576 |
| 8 | P12273 | Prolactin-inducible protein | 18 /4.8 |
| 9 | P31025 | Von Ebner’s gland protein | 18/5.0 |
| 10 | Q9NP55 | SPLUNC1 | 26.7 /5.4 |
| 11 | P02647 | Apolipoprotein AI | 26 /5.6 |
| 12 | P01591 | Immunoglobulin J chain | 18.1 /5.1 |
| 13 | P02765 | α_2_-HS-glycoprotein | 37.3 /5.3 |
| 14 | P25311 | Zn-α_2_-glycoprotein | 46.3/5.2 |
| 15 | P01009 | α_1_-antitrypsin | 44.3 /5.4 |
| 16 | P01833 | Immunoglobulin A | 90 /5.4 |
| 17 | P02790 | Hemopexin | 51.7 /6.5 |
| 18 | P02768 | Albumin | 68 /5.7 |
| 19 | P02788 | Lactoferrin | 76.1 /8.5 |
| 20 | P02787 | Transferrin | 81.4 /6.1 |
| 21 | P01857 | Immunoglobulin G | 36.1 /8.5 |
| 22 | P01834 | Immunoglobulin light chain(κ) | 11.6 /5.6 |
| 23 | P01037 | Cystatin SN | 16.4 /6.7 |
| 24 | P01884 | β_2_-microglobulin | 13.7 /6.1 |
| 25 | P05109 | Calgranulin A | 10.8 /6.5 |
| 26 | P01034 | Cystatin C | 12 /8.3 |
| 27 | P01037 | Cystatin SN | 16.4 /6.7 |
| 28 | P00695 | Lysozyme C | 14.1 /9 |
